# Effectiveness of Tai Chi on Blood Pressure, Stress, Fatigue, and Sleep Quality among Chinese Women with Episodic Migraine: A Randomised Controlled Trial

**DOI:** 10.1155/2022/2089139

**Published:** 2022-10-19

**Authors:** Shuting Wang, Longben Tian, Tongyu Ma, Yuen Ting Wong, Lin Jia Yan, Yang Gao, Dexing Zhang, Stanley Sai-Chuen Hui, Yao Jie Xie

**Affiliations:** ^1^School of Nursing, Faculty of Health and Social Sciences, The Hong Kong Polytechnic University, Hong Kong SAR, China; ^2^JC School of Public Health and Primary Care, The Chinese University of Hong Kong, Hong Kong SAR, China; ^3^Health Sciences Department, Franklin Pierce University, Rindge, NH, USA; ^4^Faculty of Social Science, The University of Hong Kong, Hong Kong SAR, China; ^5^Nethersole School of Nursing, The Chinese University of Hong Kong, Hong Kong SAR, China; ^6^Department of Sport, Physical Education and Health, Hong Kong Baptist University, Hong Kong SAR, China; ^7^Department of Sports Science and Physical Education, The Chinese University of Hong Kong, Hong Kong SAR, China; ^8^Research Center for Chinese Medicine Innovation, The Hong Kong Polytechnic University, Hong Kong SAR, China

## Abstract

The beneficial effects of Tai Chi on the cardiovascular risk profile and the migraine trigger factors among female migraineurs remain unknown. This study aimed to evaluate the effectiveness of a 12-week Tai Chi training on blood pressure (BP) and migraine-related trigger factors, including stress, fatigue, and sleep quality among Chinese women with episodic migraine. In this study, eligible Hong Kong Chinese women aged 18–65 years were randomly assigned to the Tai Chi group adopting a modified 33-short form of Yang style Tai Chi training for 12 weeks, followed by additional 12 weeks of self-practice or the waiting list control group that maintained the usual lifestyle for 24 weeks. The primary outcome was the changes in BP from the baseline to 12 and 24 weeks. The secondary outcomes included the stress level, fatigue, and sleep quality measured by the perceived stress scale (PSS), the numeric rating scale-fatigue (NRS-fatigue), and the Pittsburgh sleep quality index (PSQI), respectively. Significant between-group differences were found in systolic BP (−6.8 mmHg at 24 weeks, *P*=0.02), and a decreasing trend was significant across baseline, 12 weeks, and 24 weeks between groups (*P* < 0.05). The 12-week Tai Chi training significantly reduced the BP level and moderately improved stress level, fatigue status, and sleep quality among Chinese women with episodic migraine. Therefore, Tai Chi could be considered a promising mind-body exercise with good feasibility for migraineurs in the future. This trial is registered with registration number NCT03015753.

## 1. Introduction

Migraine is one of the most common headache disorders worldwide; it is characterised by attacks of headache and associated symptoms such as photophobia, phonophobia, somatosensory stimuli, nausea, and vomiting. According to the global burden of disease report, migraine imposed the second highest nonfatal burden in both 1990 and 2019 [1]. The prevalence of migraine in Asia was 11.3%–14.4% and 3.6%–6.7% in women and men, respectively [2]. Similarly, the reported prevalence of migraine in Hong Kong was 12.5%, with a female preponderance [3]. Migraine is significantly associated with an elevated risk of developing cardiovascular diseases [4]. Based on the Women's Health Study, a 15% increase in the risk of developing hypertension was observed in women with a history of migraine during a median follow-up of 12.2 years [5]. Among all the cardiovascular risk factors, blood pressure (BP) was possibly the most important modifiable one [6]. Therefore, preventing high BP among migraineurs is important. However, it is still unknown whether or not preventing migraine attacks could improve the BP among migraineurs.

Various trigger factors may stimulate migraine attacks. Stress and sleep disturbance have been considered the most frequent trigger factors of migraine [7, 8]. In a recent meta-analysis involving 85 retrospective studies from 1958 to 2015 with a total of 27,122 participants, stress (58%) and sleep (41%) were the most common triggers indicated by patients with migraine [9]. A longitudinal study with a total of 5,159 participants found that increasing stress was associated with increased headache frequency for all headache subtypes, particularly migraine [10]. As for sleep problems, disturbances or changes in sleeping patterns were among the most frequently cited trigger factors [11], and approximately two-thirds of migraine attacks began when the patients woke up [12]. Furthermore, chronically shortened sleep patterns were associated with more frequent and more severe migraine [11]. Meanwhile, fatigue has also been related to the precipitation of migraine [13]. In a multicentre prospective study using an electronic diary to record nonheadache symptoms of migraine [14], tiredness was the most common premonitory symptom of migraine, which accounts for 72% of the participants. Therefore, alleviating the influences of these trigger factors is important for the prophylaxis of migraine.

The standard treatment for migraine includes acute and prophylactic treatment. Acute treatment is used to rapidly and consistently alleviate the pain and associated symptoms. The first-line treatment for acute migraine includes nonsteroidal anti-inflammatory drugs, nonopioid analgesics, acetaminophen, or caffeinated analgesic combinations [15]. Meanwhile, the prophylactic treatment aims to reduce the frequency, severity, duration, and disability of patients with episodic or chronic migraine. One of the main first-line prophylactic medications is beta-blockers [16]. However, people with poorly controlled migraine attacks were at risk of a medication overdose, thereby resulting in medication-overuse headaches [17]. Serious gastrointestinal and cardiovascular side effects have also been reported. Therefore, nonpharmacological approaches are important alternatives in treating migraine. In recent decades, regular exercise has been recommended for migraine prophylaxis [17]. Mind-body exercise is often used as complementary and alternative medicine, which has been implemented by various migraineurs because of its relatively low cost, as well as low physical and emotional risk [18]. In recent years, Tai Chi, as a form of mind-body exercise, has gained popularity worldwide. Tai Chi can slowly and gently move the *q*_*i*_ (vital energy) throughout the body, thereby integrating both physical and spiritual elements. The significant beneficial effects of Tai Chi in reducing the severity of headaches and improving energy expenditure, emotional well-being, and mental health have already been reported in previous literature [19]. We believe that Tai Chi is a potential prophylactic therapy for the prevention of migraine. Moreover, Tai Chi has positive physiological and psychosocial effects on health outcomes among patients with various chronic conditions [20–22]. However, there was limited evidence evaluating its effectiveness among migraineurs. We thereby conducted a pilot study to investigate the prophylactic efficacy of Tai Chi on migraine attacks [23]. We further analysed the data in this article. As far as we know, this was the first randomised controlled trial that examined whether or not Tai Chi had beneficial effects on BP and typical migraine trigger factors in terms of stress, fatigue, and sleep quality among Hong Kong Chinese women with episodic migraine.

## 2. Methods

### 2.1. Study Design

The study was a two-arm, individual-level, parallel-group, randomised controlled trial (RCT), comprising one intervention group that received a 12-week Tai Chi training with an additional 12-weekfollow-up and one waiting list control group. The waiting list setting aimed to encourage subjects in the control group to keep in the study by providing them with delayed Tai Chi training at the end of the study (24 weeks).

This study was approved by the Human Subjects Ethics Subcommittee, the Hong Kong Polytechnic University (reference no: HSEARS20160329002). Participation was voluntary and written informed consent was collected from all participants. All collected personal information and data remained confidential. Should there be any adverse events associated with the intervention, the participants would be asked to immediately stop the intervention and seek medical attention if necessary. All adverse events would be recorded and reported to the ethics committee. All procedures were implemented based on the 1964 Declaration of Helsinki and its later amendments or comparable ethical standards.

### 2.2. Participants

#### 2.2.1. Eligible Criteria and Sample Size

The sample population of this study was Hong Kong Chinese women with clinical diagnoses of episodic migraine. Subjects were eligible if they were 18–65 years old with a diagnosis of episodic migraine (≤15 migraine days per month) based on the third edition of the International Classification of Headache Disorders (ICHD-III) [24]. They should also have at least one of the following symptoms: nausea, vomiting, photophobia, or phonophobia, and they should be able to practice Tai Chi at a designated level. Subjects would be excluded if they had secondary headaches or other neurological disorders or if they experienced Tai Chi or other mind-body exercises (e.g., yoga, biofeedback, and medication) for migraine prevention. In addition, those who received pharmacological prophylactic treatment or other alternative treatments in the past 12 weeks were excluded.

Referring to other complementary treatments [25], Tai Chi training was expected to reduce one migraine attack each month. Using PASS software, a sample of 30 in each group was needed to achieve 85% power to detect a difference of 1.0 time (standard difference, SD: 1.6) reduction in attack, with *α* = 0.050. Taking a 10% dropout rate into consideration, additional five subjects were recruited in each group to ensure that the statistical power was higher than 80%.

#### 2.2.2. Recruitment

Participants were recruited via e-mail, the school's alumni system, and WhatsApp, as well as fliers and posters disseminated in the communities. Prospective participants who met the basic criteria after the initial screening were asked to record their migraine attacks in the next 4 weeks by using a migraine diary, including the frequency, intensity, duration, and medication usage of migraine attacks. The diaries were collected for further consultation with the collaborative neurological physician at the University Health Service (UHS) to make the final diagnosis of migraine based on the ICHD-III criteria [24].

#### 2.2.3. Randomisation, Blinding, and Concealment

Eligible participants were randomly assigned to a Tai Chi training group (TC) or waiting list control group (control) in a 1 : 1 ratio by using the permuted block algorithm with a block size of four generated by a computer random number generator, blinded to the participant's identity. The allocation was concealed from the investigators until the assignments had been made. The outcome measurements were performed by research assistants who were blinded to the treatment group assignment.

#### 2.2.4. Interventions

A modified 33-short form of Yang style Tai Chi Chuan was adopted. The classical Yang-style Tai Chi Chuan consisted of 108 postures and thus needed a relatively long learning period [26]. This modified form has been recognised as a practical entry point for various beginners to learn and practice within a relatively short period. It has also been proven to be feasible and effective [27, 28]. In this study, the Tai Chi training was carried out for 12 weeks at the university's podium nearby A block, comprising three 1-hourinstructor-led sessions and two 1-hourself-practice sessions per week. Each 1-hour training session had a 10-minute brief warm-up period, a 45-minute standard Tai Chi routine activity, and a 5-minutecool-down stretching period. Tai Chi masters were recruited from the Gentle and Tranquil Tai Chi Chuan Association and were required to attend a training session to ensure that they fully understood the exact Tai Chi intervention procedures and delivered the same intervention protocols throughout the study. The Tai Chi training was delivered in a group format (15–18 participants per group). Two Tai Chi classes were arranged for the TC group; hence, that participants could flexibly attend based on their schedules. The Tai Chi movements and lesson schedules were printed in a handout to help participants to learn and practice Tai Chi.

Participants assigned to the waiting list control group just maintained their usual lifestyles for 24 weeks. Afterwards, they took Tai Chi training similar to the TC group. Both TC and control group participants received a HK$100 supermarket coupon at the baseline as a participation incentive for this study. Another HK$100 coupon was delivered to participants in the control group at 12 weeks to encourage them to complete the study to maintain a good retention rate.

#### 2.2.5. Outcome Measurement

The primary outcome was BP, including systolic and diastolic BP, which were measured by using a corrected electronic sphygmomanometer with appropriate cuffs after the participants took at least 10 minutes of seated rest. The measurements were taken twice, and the average value was used as the final measurement.

Meanwhile, the secondary outcomes were stress level, fatigue, and sleep quality. The stress level was evaluated by the 14-item perceived stress scale (PSS-14), a tool with seven positive items and seven negative items rated on a 5-point Likert scale ranging from “never” (0) to “very often” (4) to measure how often participants experience general stressfulness within a given period [29]. The PSS-14 scores were calculated by reversing the scores of the seven positive items (i.e., 0 = 4, 1 = 3, 2 = 2, 3 = 1, 4 = 0) and then summing up all 14 items. The total score on the PSS-14 ranges from 0 to 56, with higher scores indicating higher perceived stress. The adequate validity and reliability of the Chinese version of the PSS-14 have been well established [30]. The numeric rating scale-fatigue (NRS-fatigue) was used to measure fatigue level. Meanwhile, sleep quality was assessed by the Chinese version of the Pittsburgh sleep quality index (PSQI), a 19-itemself-rated questionnaire for measuring subjective sleep quality over the past months [31]. The questionnaire consists of seven clinically derived components, including sleep quality, sleep latency, sleep duration, habitual sleep efficiency, sleep disturbances, use of sleeping medications, and daytime dysfunction, each weighted equally from 0 to 3. The global score of PSQI ranges from 0 to 21, with a higher score indicating worse sleep quality. The Chinese version of PSQI has been proven to have acceptable test-retest reliability with a coefficient of 0.85 in community-dwelling Chinese adults with or without primary insomnia [32]. All the above measurements were taken at baseline, after intervention (12-week), and at the end of the follow-up period (24-week), respectively.

Data on demographics including age, marital status, education, employment, housing, living district, and family income, as well as anthropometrics (weight and height), medical history, lifestyle factors (drinking, smoking, and physical activity), reproductive information, and family history of migraine were collected in a structured interview. Weight was measured by a weighing machine to the nearest 0.1 kilograms; meanwhile, height was measured by the height measurer to the nearest 0.1 meter. Body mass index (BMI) was then calculated as weight in kilograms divided by height in meters squared. Physical activity was then measured by using the Chinese version of the International Physical Activity Questionnaire (IPAQ), which has been reported to be reliable and valid for the measurement of total physical activity in the Chinese population [33]. The results were reported as metabolic equivalent of tasks (METs) and categorised into three levels: inactive, minimally active, and health-enhancing physical activity (HEPA) active [34]. All measurements of the above variables were conducted at the baseline, 12 weeks, and 24 weeks, except for the demographics.

### 2.3. Statistical Analysis

Statistical analyses were performed by using statistic software, and SPSS 23.0 (SPSS Institute) was used for data analysis. Descriptive statistics including mean (SD) or median (interquartile range, IQR) for continuous variables, and numbers and proportions (%) for categorical variables were calculated. The paired *t*-test was used within the group comparison of normally distributed variables, and a Wilcoxon signed-rank test was used for skewed variables. An independent *t*-test was used for between-group comparison. In addition, repeated measure analysis of covariance (ANCOVA) was used to test the difference of mean changes and the interaction effect between time and group across baseline, 12 weeks, and 24 weeks, respectively. The variables which were significantly different between groups at baseline were inputted as covariates in the ANCOVA. Both per-protocol and intention-to-treat (ITT) analyses were performed. Statistical significance was defined as a *p* value <0.05 by using two-tailed tests.

## 3. Results

In total, 189 subjects initially enrolled from 2016 to 2017; 82 of them met the eligibility criteria and completed the baseline measurement and were randomly assigned to the TC group (*n* = 42) and control group (*n* = 40). After randomisation, a total of 40 participants in the TC group started the Tai Chi training, whereas the 33 participants in the control group remained in the study, and 10 of them withdrew for several reasons. A participant in the TC group also withdrew. Therefore, there were a total of 39 and 23 participants in the TC group and control group, respectively, kept in the study until the end of the 24 weeks. However, five participants in the TC group were excluded from the per-protocol analysis because they only attended less than 10 Tai Chi sessions and thus were considered invalid. The retention rates in the TC and control groups were 98% (39/40) and 70% (23/33), respectively. There were no significant differences in the baseline characteristics between the participants who completed the whole study and those who withdrew (all *P* > 0.05). The flow diagram of the study is shown in Supplementary Materials (available here).

### 3.1. Baseline Characteristics

The baseline characteristics of the two groups are presented in Table 1. The average age of the participants was 50.9 and 47.1 in the TC group and control group, respectively. More than 80% of participants were inactive or minimally active in physical activity. More participants in the control group had a family history of migraine; however, there was no significant difference between the two groups. The majority of participants lived in Kowloon and New Territories. In addition, all participants were nonsmokers. The only difference was observed in the drinking participants in the control group, which indicated they were more likely to drink than those in the TC group (*P* < 0.05). Moreover, it showed that diastolic BP in the control group was significantly higher than those in the TC group (*P* < 0.05). No significant differences were found in other variables.

### 3.2. Primary Outcome: Changes in Blood Pressure before and after Intervention and the Between-Group Differences

Table 2 shows the change of outcome measurements from the baseline to 12 weeks and 24 weeks in the TC group and control group. Variables with significant differences between groups at baseline were adjusted as covariates. A significant decrease in systolic BP was observed in the TC group with a mean change of −5.7 (−9.1 to −2.3) mmHg based on the ITT analysis; meanwhile, the control group did not show any significant changes in BP at 12 weeks (*P* > 0.05). In addition, both TC and control groups had a significant decrease in systolic and diastolic BP at 24 weeks as compared with the baseline, and the reduction of systolic BP in the TC group was significantly greater than that in the control group (*P* < 0.05). In the per-protocol analysis, similar results were found in BP, except a 3.0 mmHg (95% confidence interval, CI: −5.5 to −0.5 mmHg) reduction in diastolic BP was found in the TC group at 12 weeks. By the repeated ANCOVA analysis, systolic BP in the TC group was significantly lower at 24 weeks and across the whole study period (both *P* < 0.05) than in the control group (Table 3).

### 3.3. Secondary Outcomes: Changes in PSQI Score, PSS Score, and NRS-Fatigue Score before and after Intervention and the Between-Group Differences

The sleep quality in the TC group significantly improved as compared with the baseline, with a 1.4-unit decrease in the PSQI score (*P* < 0.01) at 12 weeks. The improvement of sleep quality in the TC group was greater (*P* < 0.01), and significant alleviations in stress and fatigue were also found among participants in the TC group at 24 weeks. The PSS score and NRS-fatigue score in the TC group were reduced by −2.0 (95% CI: −3.7 to −0.2) and −1.2 (95% CI: −1.9 to −0.4), respectively. There were no significant changes found in stress level, fatigue, and sleep quality in the control group (all *P* > 0.05), and the per-protocol analysis suggested similar results (Table 2).

## 4. Discussion

This is the first study to evaluate the beneficial effects of Tai Chi on reducing BP and migraine-trigger factors among Chinese women with episodic migraine. As expected, we found that participants in the TC group reduced their BP, alleviated stress and fatigue, and improved sleep quality after a 12-week Tai Chi training and 12-weekfollow-up. The significant difference between groups was only found in the systolic BP as compared with the control group.

The beneficial effects of Tai Chi in reducing BP have been reported in previous studies with various Tai Chi regimens [35, 36]. In RCTs that adopted a 12-week Tai Chi practice and were conducted in the Chinese population, a greater BP reduction (10–19 mm Hg reduction in systolic BP and 7–9 mm Hg reduction in diastolic BP) was reported [37, 38]. This could be because the majority or all of the participants in these previous studies had high-normal BP or hypertension. In addition, it is expected to observe a more significant BP change in hypertensive patients than in subjects with normal BP. In our study, a more significant reduction in BP was observed at 24 weeks, which may be due to the effect of voluntary, continuous TC practice after the 12-week intervention, as the participants still kept 1.5 times of practice per week and 20 min for each practice at the end of 24 weeks. Meanwhile, similar BP‐lowering effects have been reported with other mind‐body therapies. For example, an RCT involving a 12-week yoga program observed a significant 6 and 5 mmHg reduction in 24-hour systolic and diastolic BP in adults with untreated prehypertension or stage 1 hypertension [39]. The mechanism of mind-body exercise in the control of BP remains unclear. However, previous studies suggested that it could be due to improved baroreceptor sensitivity or decreased sympathetic drive [40, 41].

Although a limited number of studies have examined the effectiveness of Tai Chi on stress level, fatigue, and sleep quality in migraineurs, existing evidence suggests that Tai Chi is an effective complementary and alternative approach to improve psychological well-being and sleep quality in patients with other chronic conditions. Meta-analyses have shown that Tai Chi could significantly reduce stress, enhance mood, and improve fatigue in patients with chronic conditions, including cancer, rheumatoid arthritis, depression, and so on, as compared with conventional treatment (e.g., medical consultant and health education) or low-effect exercise (e.g., stretching and walking) [42, 43]. Similarly, Tai Chi significantly improved sleep quality as assessed by the global PSQI score among healthy older adults and patients with chronic conditions [44–46]. This previous evidence coincides with our findings that Tai Chi significantly alleviated stress and fatigue and improved sleep quality among migraineurs in the TC group. These finds revealed that, in the future, Tai Chi holds the potential to be a safe and effective treatment for mental and psychological disorders, especially combined with phytotherapy therapies [47, 48]. However, the difference was not statistically significant as compared with the control group, which may be due to the short duration of the intervention and the small sample size.

Although other common nonpharmacologic treatments, such as biofeedback, cognitive-behavioural therapy, and acupuncture, have been reported to be effective for the prophylaxis of migraine [49–51], Tai Chi is more economic-friendly and flexible for migraineurs as this can be performed by themselves at home, unlike biofeedback and cognitive-behavioural therapy which usually include counselling or acupuncture which is often provided by physicians and the effectiveness varies over various factors (e.g., treatment settings and patients' attitude and expectations) [52]. However, the underlying mechanisms of the benefits of Tai Chi on stress, fatigue, and sleep quality are less examined. It is possibly because Tai Chi, as a body-mind exercise, has incorporated a structured cognitive component, which is also referred to as meditation [53]. With this structured cognitive component, the practitioner's mind directs the body in performing the movements, which makes it more effective in bringing both physiological and psychological benefits to the practitioners [54]. In addition, slow movements in Tai Chi make it easier to practice for migraineurs without causing harm or side effects. Therefore, our findings on Tai Chi are proven safe and effective alternative prophylaxis approaches for migraineurs.

However, this study has some limitations. First, it is impossible to implement blinding to participants, given that Tai Chi is a behaviour-based intervention. In addition, the waiting list control group was utilised in this study to maintain a high retention rate. Therefore, the placebo effects might not be fully examined or the effect could be slightly overestimated. Nevertheless, we believe that the evidence from this pilot study is still acceptable and reliable because the intervention design was rigorously scientific, and the effect detected existed. An active control group is suggested for further studies. The findings from this study can be used as a foundation for further investigations. Second, measurements used in this study for stress level, fatigue, and sleep quality were subjective, which may have recall bias. However, the measurement scales used in this study were all widely used tools with acceptable reliability and validity, and it is unlikely to cause a significant effect on the resulting outcomes. Third, the relatively small sample size might reduce the strength of the study. Therefore, future study with larger sample size and longer intervention period is needed to further evaluate the clinical efficacy of Tai Chi training on certain outcomes.

## 5. Conclusion

The 12-week Tai Chi training could be recommended as a promising mind-body exercise for Chinese female migraineurs because Tai Chi has beneficial effects on lowering BP, alleviating stress, reducing fatigue, and improving sleep quality. The finding provided new insight regarding the role of mind-body exercise in improving health conditions among migraineurs. The modified short-form Tai Chi is easy to learn, and its practice is cost-effective and convenient. It would be helpful for healthcare and medical practitioners to make an evidence-based suggestion, which has the potential to be promoted and widely implemented in community settings.

## Figures and Tables

**Table 1:** Baseline characteristics and migraine trigger factors of the participants^a^.

	Intervention (*n* = 40)	Control (*n* = 33)
Age, years		
<45	8 (20.0)	13 (39.4)
≥45	32 (80.0)	20 (60.6)

BMI, kg/m^2^		
Nonobese (<23)	21 (52.5)	17 (51.5)
Overweight/obesity (≥23)	19 (47.5)	16 (48.5)
Systolic blood pressure, mmHg	123.3 (15.3)	116.9 (14.2)
Diastolic blood pressure, mmHg	76.8 (10.5)	71.7 (9.1)
Stress level (PSS score)	25.1 (6.7)	24.5 (6.2)
Fatigue (NRS-fatigue score)	5.8 (2.0)	6.0 (1.7)
Sleep quality (PSQI score)	8.5 (3.6)	7.8 (3.2)

Marital status		
Single	14 (35.0)	6 (18.2)
Married/cohabitating/divorced/separated/widowed	26 (65.0)	27 (81.8)

Education		
Matriculation or below	23 (57.5)	16 (48.5)
Tertiary or above	17 (42.5)	17 (51.5)

Occupation		
Employed	21 (52.5)	22 (66.7)
Unemployed	13 (32.5)	10 (30.3)
Retired	6 (15.0)	1 (3.0)

Housing		
Self-owned property	26 (65.0)	20 (60.6)
Rental property and others	13 (32.5)	13 (39.4)

Living district		
Hong Kong Island	2 (5.0)	6 (18.2)
Kowloon	18 (45.0)	12 (36.4)
New territories	19 (47.5)	15 (45.5)

Monthly family income		
<$40,000	28 (70.0)	21 (63.6)
≥$40,000	11 (27.5)	11 (33.3)
Hypertension^b^	2 (5.0)	6 (18.2)
High cholesterol^b^	8 (20.0)	3 (9.1)
Health check in the past 2 years^b^	27 (67.5)	22 (66.7)
Clinical breast examination in the past 2 years^b^	13 (32.5)	12 (36.4)
IPAQ total physical activity, MET-min/week	1726.5 (1019.6–2556.8)	2302.0 (1044.8–5625.0)

Physical activity category		
Inactive	6 (15.0)	2 (6.1)
Minimally active	29 (72.5)	26 (78.8)
Health-enhancing physical activity active	3 (7.5)	5 (15.2)
Drinking^b^	21 (52.5)	25 (75.8)
Age of menarche, year(s)	12.3 (1.6)	12.9 (1.6)
Length of the current menstrual cycle, day(s)^c^	28.9 (2.0)	30.0 (4.7)
Menopause^b^	23 (57.5)	13 (39.4)
Family history of migraine^b^	11 (27.5)	14 (42.4)

^a^Values reported as mean (SD), *n* (%), or median (interquartile range) for each group where appropriate. ^b^Variables showed the number and percentage that counted “Yes” for each group. ^c^Among those who had not started menopause only (*n* = 31).

**Table 2:** Changes in blood pressure, stress, fatigue, and sleep quality from baseline to 12 weeks and 24 weeks.

Outcome^a^	ITT		Per-protocol^c^			
	Intervention (*n* = 40)	Control (*n* = 33)	*P* ^b^	Intervention	Control	*P* ^b^
Systolic blood pressure (mmHg)						
12 weeks	117.6 (17.2)	114.3 (13.2)	0.368	117.6 (16.9)	114.5 (13.8)	0.426
24 weeks	112.5 (15.6)	112.9 (12.8)	0.915	113.0 (14.5)	113.0 (12.9)	0.986
Mean change from baseline to 12 weeks	−5.7 (−9.1 to −2.3)^∗∗^	−2.5 (−5.6 to 0.5)	0.828	−6.6 (−10.6 to −2.6)^∗∗^	−2.6 (−5.8 to 0.6)	0.398
Mean change from baseline to 24 weeks	−10.8 (−14.3 to −7.4)^∗∗^	−4.0 (−7.3 to −0.7)^∗^	0.020	−11.2 (−15.2 to −7.3)^∗∗^	−3.4 (−7.5 to 0.7)	0.079

Diastolic blood pressure (mmHg)						
12 weeks	74.7 (10.2)	70.1 (9.1)	0.049	74.1 (9.8)	70.3 (9.5)	0.124
24 weeks	71.9 (10.7)	68.0 (10.1)	0.120	72.3 (10.2)	68.3 (10.7)	0.169
Mean change from baseline to 12 weeks	−2.1 (−4.7 to 0.5)	−1.7 (−4.3 to 1.0)	0.517	−3.0 (−5.5 to −0.5)^∗^	−1.5 (−4.2 to 1.3)	0.887
Mean change from baseline to 24 weeks	−4.9 (−7.2 to −2.5)^∗∗^	−3.7 (−6.5 to −0.9)^∗^	0.721	−4.8 (−7.4 to −2.3)^∗∗^	−2.5 (−6.2 to 1.6)	0.733

Stress						
12 weeks	23.5 (6.3)	25.4 (8.1)	0.280	23.2 (5.8)	24.7 (7.9)	0.376
24 weeks	23.1 (7.4)	24.2 (8.1)	0.541	22.5 (6.9)	23.7 (7.0)	0.549
Mean change from baseline to 12 weeks	−1.6 (−3.1 to 0.0)	0.9 (−1.0 to 2.8)	0.085	−1.7 (−3.5 to 0.1)	0.2 (−1.4 to 1.9)	0.068
Mean change from baseline to 24 weeks	−2.0 (−3.7 to −0.2)^∗^	−0.2 (−2.8 to 2.3)	0.223	−2.4 (−4.4 to −0.4)^∗^	−1.6 (−4.4 to 1.2)	0.411

Fatigue						
12 weeks	5.4 (2.0)	5.7 (2.1)	0.542	5.2 (2.0)	5.7 (2.1)	0.355
24 weeks	4.6 (2.3)	5.4 (2.1)	0.433	4.2 (2.0)	5.6 (2.1)	0.021
Mean change from baseline to 12 weeks	−0.3 (−0.9 to 0.3)	−0.3 (−1.0 to 0.4)	0.989	−0.3 (−1.0 to 0.4)	−0.3 (−1.1 to 0.4)	0.867
Mean change from baseline to 24 weeks	−1.2 (−1.9 to −0.4)^∗∗^	−0.7 (−1.4 to 0.0)	0.560	−1.3 (−2.1 to −0.4)^∗∗^	−0.6 (−1.4 to 0.3)	0.301

Sleep quality						
12 weeks	7.1 (3.6)	7.8 (3.8)	0.455	6.8 (3.7)	7.4 (3.4)	0.476
24 weeks	6.9 (3.2)	7.5 (3.6)	0.433	6.8 (3.3)	7.5 (3.0)	0.470
Mean change from baseline to 12 weeks	−1.4 (−2.3 to −0.5)^∗∗^	−0.1 (−1.2 to 1.1)	0.097	−1.9 (−2.8 to −0.9)^∗∗^	−0.2 (−1.4 to 1.0)	0.046
Mean change from baseline to 24 weeks	−1.7 (−2.7 to −0.7)^∗∗^	−0.4 (−1.5 to 0.8)	0.220	−1.8 (−2.9 to −0.7)^∗∗^	−0.7 (−1.7 to 0.3)	0.133

^a^Values are presented as mean (SD) for 12-week and 24-week measurements; mean (95% CI) for mean change from the baseline. ^b^*P* values generated from the independent *t*-test or Wilcoxon signed-rank test were appropriate. ANCOVA was used to compare the difference in mean changes between groups, and variables with significant differences between groups at the baseline were adjusted as covariates. ^c^Per-protocol at 12 weeks: intervention (*n* = 33), control (*n* = 30); at 24 weeks: intervention (*n* = 33), control (*n* = 23). ^*∗*^*P* < 0.05 generated from within-group comparison by the paired *t*-test were appropriate. ^*∗∗*^*P* < 0.01 generated from within-group comparison by the paired *t*-test were appropriate.

**Table 3:** Between-group differences in blood pressure, stress, fatigue, and sleep quality at 12 weeks and 24 weeks.

Outcome^a^	Between-group differences at 12 weeks		Between-group differences at 24 weeks		
	Intervention vs. control	*P* ^b^	Intervention vs. control	*P* ^b^	*P* ^c^
Systolic blood pressure (mmHg)					
ITT	−3.2 (−7.8 to 1.4)	0.828	−6.9 (−11.6 to −2.1)	0.020	0.020
Per-protocol	−4.0 (−9.1 to 1.1)	0.398	−7.9 (−13.6 to −2.1)	0.079	0.216

Diastolic blood pressure (mmHg)					
ITT	−0.4 (−4.2 to 3.3)	0.706	−1.2 (−4.7 to 2.4)	0.296	0.524
Per-protocol	−1.5 (−5.2 to 2.1)	0.309	−2.3 (−6.5 to 1.9)	0.258	0.387

Stress					
ITT	−2.4 (−4.8 to −0.0)	0.085	−1.7 (−4.7 to 1.2)	0.223	0.222
Per-protocol^d^	−2.0 (−4.4 to 0.4)	0.068	−0.8 (−4.1 to 2.4)	0.411	0.214

Fatigue					
ITT	−0.0 (−0.9 to 0.9)	0.989	−0.5 (−1.5 to 0.5)	0.560	0.795
Per-protocol	0.0 (−1.0 to 1.1)	0.867	−1.1 (−1.2 to 1.0)	0.301	0.583

Sleep quality					
ITT	−1.3 (−2.7 to 0.1)	0.097	−1.3 (−2.8 to 0.2)	0.220	0.255
Per-protocol	−1.7 (−3.2 to −1.2)	0.298	−1.1 (−2.7 to 0.4)	0.133	0.200

^a^Values are presented as mean (95% CI) for mean change between groups. ^b^*P* values were calculated for the time × group interaction effects from baseline to 12 weeks or from the baseline to 24 weeks between groups by repeated ANCOVA; variables with significant differences between groups at the baseline were adjusted as covariates. ^c^*P* values were calculated for the time × group interaction effects across the baseline, 12 weeks, and 24 weeks between groups by repeated ANCOVA; variables with significant differences between groups at the baseline were adjusted as covariates.

## Data Availability

The data used to support the findings of this study are available from the corresponding author upon request.

## Supplementary Materials

Supplementary Materials Supplementary Figure I: The flow diagram of the study. CONSORT 2010 checklist: Information to include when reporting a randomised trial.
