# Supplementary figures and images for: Effectiveness of Tai Chi on Blood Pressure, Stress, Fatigue, and Sleep Quality among Chinese Women with Episodic Migraine: A Randomised Controlled Trial
Source: Evid Based Complement Alternat Med. 2022 Oct 19;2022:2089139. doi: 10.1155/2022/2089139 (PMC9605843; doi:10.1155/2022/2089139)

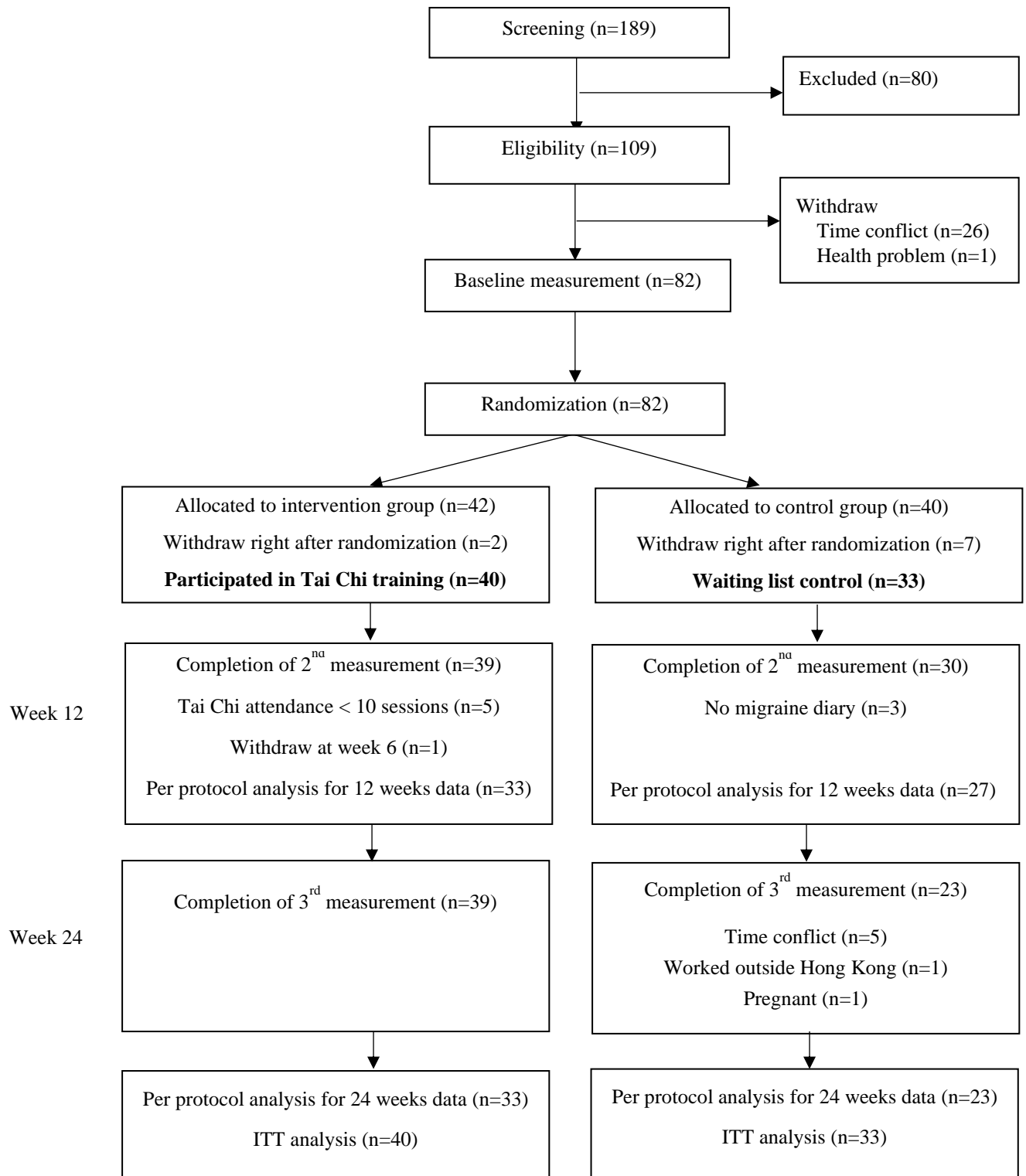

Supplementary Figure I. Flow diagram of the whole RCT

Supplement: Supplementary Materials — Supplementary Figure I: The flow diagram of the study. CONSORT 2010 checklist: Information to include when reporting a randomised trial. [file 2089139.f1.zip › Supplementary Figure I.pdf]
